# Incidence of Retinal Complications in a Cohort of Newly Diagnosed Diabetic Patients

**DOI:** 10.1371/journal.pone.0100283

**Published:** 2014-06-25

**Authors:** Elisa Martín-Merino, Joan Fortuny, Elena Rivero-Ferrer, Luis Alberto García-Rodríguez

**Affiliations:** 1 Centro Español de Investigación Farmacoepidemiológica, Madrid, Spain; 2 DS&E - Global Clinical Epidemiology, Novartis Farmaceutica S.A., Barcelona, Spain; University of Michigan Medical School, United States of America

## Abstract

**Purpose:**

We aimed at estimating the incidence of diabetic retinopathy (DR) and maculopathy (DMP) among newly diagnosed type 1 (t1DM) and type 2 diabetic patients (t2DM) in the United Kingdom primary care system. The incidence of DMP among patients with DR was also estimated.

**Method:**

We conducted a cohort study using The Health Improvement Network database. The cohort included 64,983 incident diabetic patients (97.3% were t2DM) aged 1–84 years diagnosed between 2000 and 2007. This cohort was followed from the date of diabetes diagnosis until recording of DR or DMP in two separate follow-ups. Follow-up was censored at 85 years of age, death, or end of 2008. An additional follow-up was conducted from DR to DMP diagnosis using similar censoring reasons. DR and DMP cumulative incidences were calculated as well as incidence rates (IR; cases per 1,000 person-years) per calendar period (2000–2001 and 2006–2007).

**Results:**

Follow-up for DR: 9 years after diabetes diagnosis, 28% of t2DM and 24% of t1DM patients had developed DR (7,899 incident DR cases). During the first 2 years with diabetes, the IR was almost 2 times higher in patients diagnosed with diabetes in 2006–2007 (47.7) than among those diagnosed in 2000–2001 (24.5). Follow-up for DMP: 9 years after diabetes diagnosis, 3.6% of t2DM and 4.4% of t2DM patients had developed DMP (912 incident DMP cases). During the first 2 years with diabetes, the IR was three times higher in patients diagnosed with diabetes in 2006–2007 (5.8) than among those diagnosed in 2000–2001 (1.8). Macular oedema occurred in 0.8% of patients.

**Conclusions:**

In a cohort of incident diabetes, 28% of patients developed retinopathy and 4% maculopathy within the first 9 years. The 2-year IRs of DR and DMP were higher in patients diagnosed with diabetes during the period 2006–2007 than in those diagnosed during the 2000–2001 period.

## Introduction

Diabetic retinopathy is the first cause of blindness in the working age population in the United Kingdom (UK) [1]. Despite the implementation of strict protocols for managing diabetes in developed countries, a high prevalence of diabetic ocular microangiopathy still exists. In the Liverpool Diabetic Eye Study it was observed that during the first screening for retinopathy in diabetic patients diagnosed during 1991–1999, 25–39% had DR at the time of diabetes diagnosis [2], [3]. It has been estimated that 38% of type 2 diabetic patients and 45% of type 1 diabetic patients will develop DR in a 6-year period [2], [3]. Few studies have documented the risk of retinal complications after the implementation of systematic programs for the prevention of diabetes complications in the last decade [4].

The aim of the present study was to assess the incidence of retinopathy and maculopathy in a UK population of incident diabetic patients registered in the UK primary care database THIN (The Health Improvement Network), overall, by type of diabetes and calendar period. We also estimated the incidence of maculopathy after initial DR diagnosis.

## Methods

### Ethics statement

The study research protocol was approved by the UK Research Ethics Committee (09/H0305/64).

### Source of data

The Health Improvement Network is a longitudinal primary care medical records database containing anonymized data on over 3 million active patients currently registered with participating UK primary care practices [5]. These patients are representative of the entire UK population with respect to demographics and prevalence of major conditions [6]. THIN database contains individual patient information recorded by primary care practitioners (PCPs) as part of their routine clinical care such as demographic factors, PCP consultations, referrals, hospitalizations, laboratory test results, and prescriptions written by PCPs. Letters from specialist visits and hospital admissions (i.e., discharge letters) are also available. Diagnoses and test procedures are recorded using READ codes [7], [8]. Prescriptions written by PCPs are generated and coded automatically in the database using the Multilex drug dictionary [9].

### Ascertainment of incident diabetic cohort

**Figure 1:**
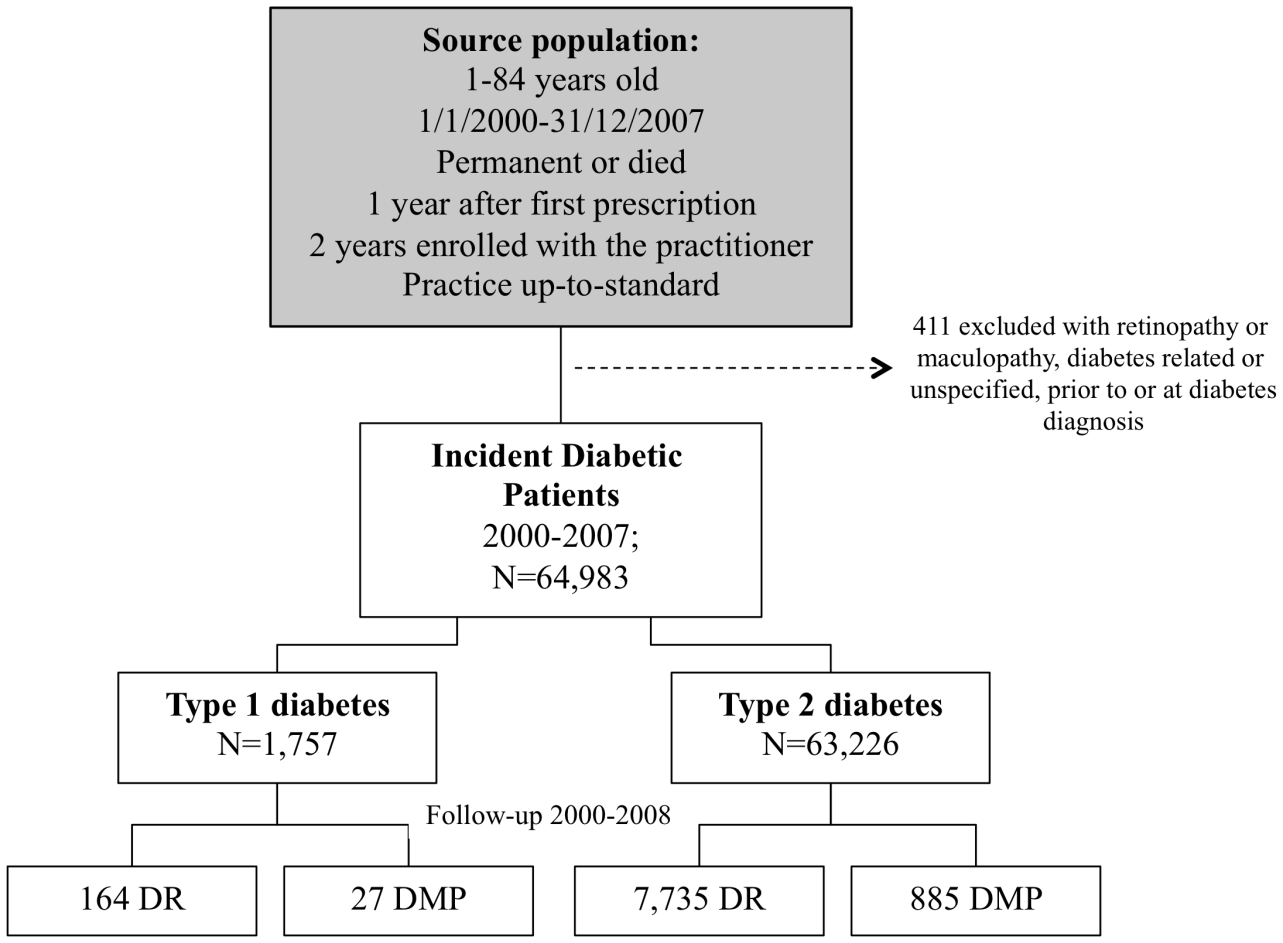
Flow of participants in cohort study of incidence of diabetic retinopathy (DR) and maculopathy (DMP) in patients with newly diagnosed type 1 and type 2 diabetes.

Detailed information on ascertainment of the study cohort has been reported elsewhere [10]. Briefly, we identified a cohort of newly diagnosed diabetic patients aged 1–84 years between January 2000 and December 2007 (Figure 1). The date of diabetes onset was defined as the first recorded diabetes diagnosis or prescription of antidiabetic treatment. This date was used as start date of follow-up to ascertain DR and DMP diagnosis. We ascertained diabetes type based on type-specific READ codes. When no diabetes type was recorded or when there was a conflict between the recorded type and the patient characteristics, we classified the diabetes type according to age at diagnosis, antidiabetic treatment, and BMI. A similar definition for diabetes type was described elsewhere by our team [11].

We excluded from the study cohort all patients with a diagnosis code for retinopathy or maculopathy, whether diabetes related or unspecified, recorded any time before or on the same date of the first diagnosis of diabetes (N = 411) [10].

### Ascertainment of diabetic retinopathy and maculopathy cases

We identified DR cases as patients with a recorded diagnostic code compatible with a diagnosis of retinopathy related to diabetes. Diagnostic codes of retinopathy not related to diabetes were excluded. We identified DMP cases as patients with a recorded code suggesting a diagnosis of maculopathy related to diabetes including macular oedema, exudative maculopathy or any other non-specific maculopathy code.

READ codes used for DR and DMP ascertainment and the validation process of diagnoses using patient's electronic medical records and questionnaires sent to the PCPs have been described previously. Confirmation rates were 78.0% for DR diagnosis codes and 78.8% for DMP diagnosis codes [10].

We prospectively followed the incident diabetic cohort from start date until first recording of DR or DMP diagnosis [10] in two separate follow-ups (Figure 1). In the first follow-up, patients were censored at the earliest occurrence of any of the following endpoints: DR diagnosis, 85 years of age, death, or 31^st^ December 2008. In the second follow-up, patients were censored at the earliest occurrence of any of the following endpoints: DMP diagnosis, 85 years of age, death, or 31^st^ December 2008. In addition, we followed the subset of patients who developed DR during the first follow-up, from the date of DR diagnosis until the first diagnosis of DMP, 85 years of age, death, or 31 December 2008, whichever occurred first. All DMP cases were manually reviewed to identify subjects with macular oedema (DMO).

### Incidence estimation

Cumulative incidences of DR and DMP among diabetic patients, as well as their confidence intervals, were calculated by diabetes type using the Kaplan-Meier method [12]. Incidence rates (IR) for DR and DMP identified during the first 2 years after diabetes diagnosis, were calculated among patients newly diagnosed with diabetes in two distinct periods corresponding to the beginning (2000–2001) and end (2006–2007) of the study period. IRs were computed dividing the total number of cases of each outcome by the corresponding total number of person-years (p-y) of follow-up assuming the Poisson distribution [13]. Statistical package Stata/IC version 11.1 was used for all statistical analyses [14].

## Results

There were 64,983 incident diabetic patients of whom 97.3% were type 2 and 2.7% type 1 diabetics (Figure 1). The mean age at diabetes onset was 61.3 years (range 6–84) for patients with type 2 diabetes and 19.1 years (range 1–40) for patients with type 1 diabetes. The majority of diabetic patients were men: 55.1% among type 2 diabetic patients and 60.7% among type 1 diabetic patients.

Among type 2 diabetic patients, 61% did not receive any hypoglycaemic drug during the first three months after initial diagnosis, 36% were prescribed only oral antidiabetic drugs, and 3% received insulin with or without oral antidiabetic drugs. Among type 1 diabetic patients, the corresponding percentages were 8%, 3%, and 89%. Only 7 patients classified as having type 1 diabetes by the computer-based diagnostic algorithm [11] did not receive insulin during the follow-up.

During the period 2000–2001 there were 10,645 incident diabetic patients diagnosed of whom 97.2% had type 2 diabetes, 56.3% were men, and the mean age at diabetes onset was 60.6 years (range 1–81 years). During the period 2006–2007 there were 19,569 incident diabetic patients diagnosed of whom 97.2% had type 2 diabetes, 55.5% were men, and the mean age at diabetes onset was 59.4 years (range 1–84 years). Among diabetic patients diagnosed in 2000–2001, 35% were prescribed only oral antidiabetic drugs during the first three months after initial diagnosis, and 5% received insulin with or without oral antidiabetic drugs. Among those diagnosed in 2006–2007, the percentages were 36% and 5%, respectively.

### Follow-up for diabetic retinopathy

**Figure 2:**
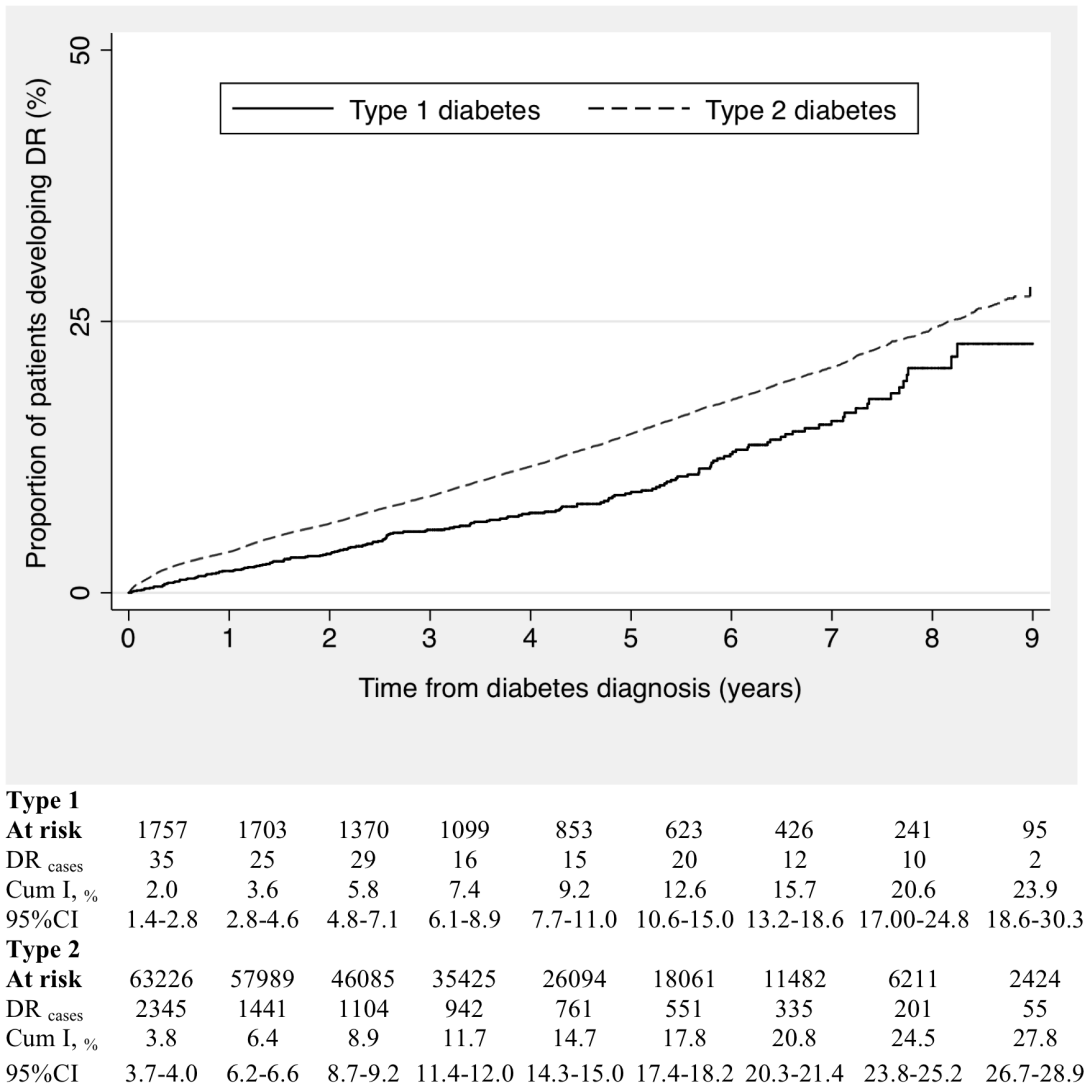
Kaplan-Meier curves and table of annual cumulative incidence of diabetic retinopathy from the date of first diabetes diagnosis recorded.

Over a median follow-up of 3.4 years we identified 7,899 patients with incident DR (7,735 among type 2 diabetes and 164 among type 1 diabetes). The mean age at DR diagnosis was 63.3 years (range 17–84) for patients with type 2 diabetes and 28.2 years (range 6–47) for patients with type 1 diabetes. The cumulative incidence of DR increased with increasing age at diagnosis of diabetes up to 40 years in both types of diabetes and remained stable thereafter for type 2 diabetics (data not shown). In the first year of follow-up, 3.8% of patients with type 2 diabetes and 2.0% of patients with type 1 diabetes had developed DR (majority during the first three months, 1.6% of patients). The crude cumulative incidences of DR were higher in type 2 than in type 1 diabetes throughout the follow-up. After 9 years, the cumulative incidences were 27.8% and 23.9% for type 2 and type 1 diabetics, respectively (Figure 2). The incidence rate of DR in the first two-years following diabetes diagnosis was 24.5 (95%CI: 21.5–27.9) per 1,000 p-y among diabetic patients diagnosed in the period 2000–2001 and 47.7 (95%CI: 44.6–50.9) per 1,000 p-y among those diagnosed in the period 2006–2007.

Among the patients with incident DR, 548 had a new diagnosis of DMP on the same day (N = 430) or before (N = 118) the diagnosis of DR. Among patients free of DMP at DR diagnosis date, we identified 318 individuals with a diagnosis code of DMP during a median follow-up time of 1.9 years. At the end of follow-up the cumulative incidences were 12.1% and 18.8%, respectively.

### Follow-up for diabetic maculopathy

**Figure 3:**
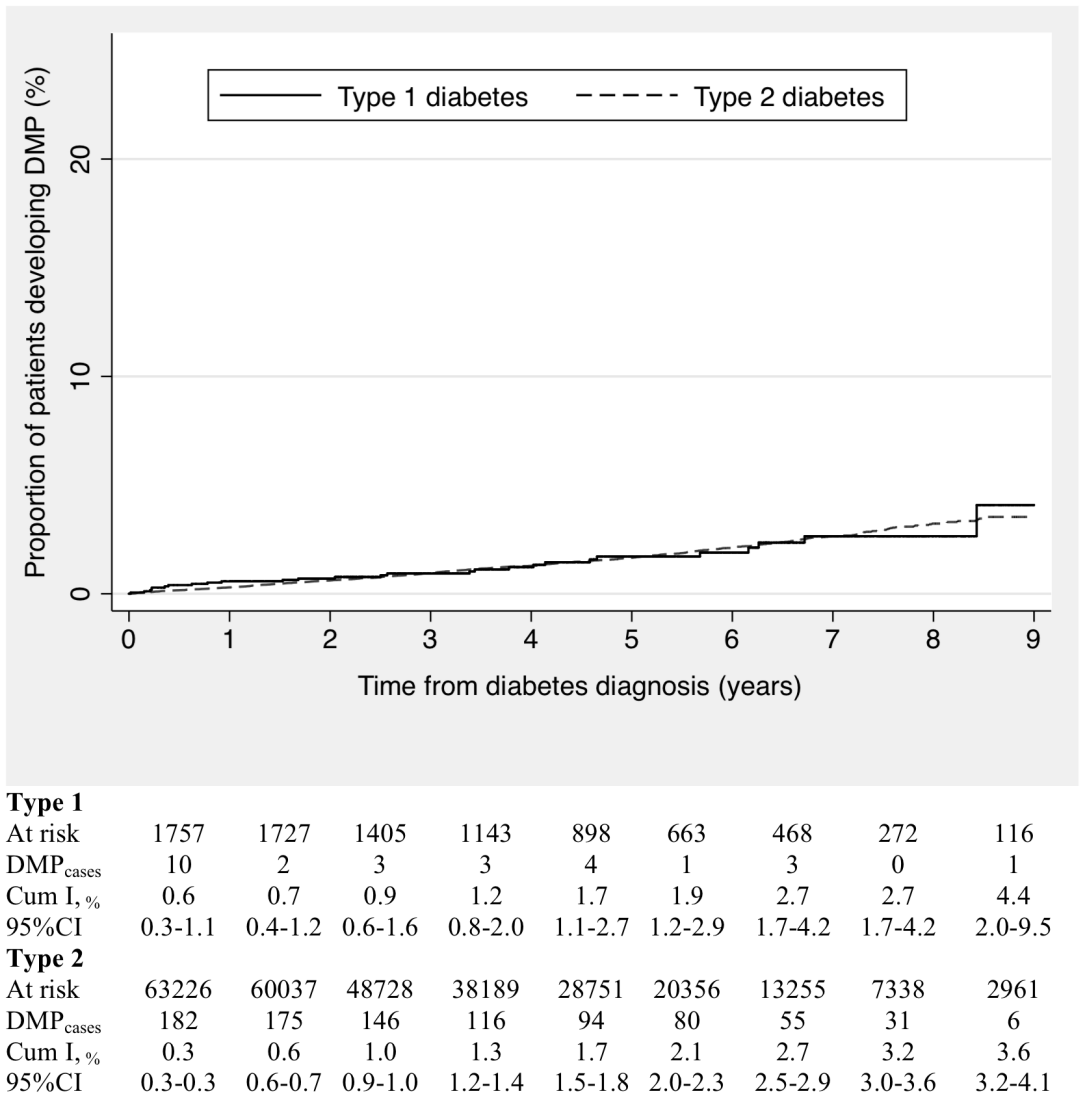
Kaplan-Meier curves and table of annual cumulative incidence of diabetic maculopathy from the date of first diabetes diagnosis recorded.

Over a median follow-up of 3.7 years, we identified 912 patients with incident DMP (885 among type 2 diabetes and 27 among type 1 diabetes). The mean age at DMP diagnosis was 62.8 years (range 20–84) for type 2 diabetics and 34.0 years (range 14–45) for type 1 diabetics. The cumulative incidence increased with increasing age at diabetes diagnosis until 40 years of age and stabilized thereafter (data not shown). After the first year of follow-up, 0.3% of patients with type 2 diabetes and 0.6% of patients with type 1 diabetes had developed DMP (0.1% of patients during the first three months). After 9 years of follow-up, the cumulative incidences were 3.6% and 4.4%, respectively (Figure 3). The incidence rate of DMP in the first two-years following diabetes diagnosis was 1.8 (95%CI: 1.0–2.9) per 1,000 p-y among diabetic patients diagnosed in the period 2000–2001 and 5.8 (95%CI: 4.8–7.0) per 1,000 p-y among those diagnosed in the period 2006–2007.

An additional analysis was conducted to identify patients with diabetic macular oedema (DMO). Among DMP cases, 215 could be specifically classified as macular oedemas (211 occurring in type 2 diabetics). Overall, at the end of follow-up 0.8% of patients had developed DMO. However, according to the previous validation study [10], 54% of the real DMO cases (estimated N = 252 DMO) would be underestimated. According to this, among the overall 912 DMP cases identified, 467 (215+252) would be estimated to be real DMO cases. This resulted in a corrected overall IR of DMO of 1.80 per 1,000 p-y (95%CI: 1.64–1.98).

## Discussion

In this cohort study performed in a primary care UK population between 2000 and 2008, we observed that among incident diabetic patients free of retinopathy at baseline, about one fourth developed diabetic retinopathy and close to 4% developed maculopathy within 9 years after initial diabetes diagnosis. Incidence increased with age until the fourth decade of life. Crude annual cumulative incidences for DR were consistently higher in type 2 than type 1 diabetic patients, while they were similar for DMP.

We estimated a 9-year cumulative incidence of DR of 28% in type 2 and 24% in type 1 diabetic patients. These incidences appear to be lower than those reported in the literature. The Liverpool Diabetic Eye Study found that the number of screened diabetic patients affected with any DR reached 38% among type 2 and 45% among type 1 diabetic patients after 6 years of follow-up from the first retina screening [2], [3]. Similarly, a study conducted on prevalent type 2 diabetic patients in the UK found a 4-year cumulative incidence of DR of 36% [15]. Finally, in a study assessing type 2 diabetic patients, Jones et al. found that around 62% had developed non-proliferative DR, 14% pre-proliferative DR, and 1% proliferative retinopathy after 9 years of disease follow-up [4].

In our study, we estimated a 9-year cumulative incidence of DMP of 3.6% in type 2 and 4.4% in type 1 diabetic patients. We observed a cumulative incidence of 0.8% of DMO in 9 years. However, according to our validation process [10], this estimation would reflect only about half the total actual DMO cases in our study. Similarly to DR, these incidences appear to be lower than those reported in the literature. The Liverpool Diabetic Eye Study reported sight-threatening maculopathy in almost 5% of type 2 and 3% of type 1 diabetic patients after 6 years of follow-up from the first retina screening [2], [3]. In the study published by Jones et al, sight-threatening maculopathy was reported to occur in 1% of patients after 9-years with diabetes [4]. A study by Klein et al. reported a higher incidence of DMO, i.e.15.8% in older-onset and 23.8% in younger-onset diabetic patients diagnosed between 1979–1980 after 9–10 years with the disease [16].

Finally, among patients with retinopathy at baseline, we found a cumulative incidence of DMP of 12.1% in type 2 and 18.8% in type 1 diabetes within 9 years. In contrast, Younis et al. found that among those with background or mild pre-proliferative DR at baseline, sight-threatening maculopathy was diagnosed respectively in 27% and 52% after 6 years in type 2 diabetes [2], and 29% and 85% after 6 years among type 1 diabetes [3]. Also, the UK Prospective Diabetes Study reports that among patients with more severe retinopathy at baseline, photocoagulation therapy –that can be seen as a proxy of the presence of maculopathy- had been prescribed in 32% of subjects after a 9-year follow-up period [17].

Several differences between our study and previous studies of DR and DMP need to be taken into account when comparing results. In our study, the follow-up was conducted on newly diagnosed diabetic patients whereas other studies investigated prevalent diabetic patients with no evidence of retinopathy at baseline [2], [3], [15]. The latter would tend to have a higher risk of developing DR and DMP during follow-up because of time elapsed since diabetes diagnosis. Also, the possibility of differences between studies in the techniques used to diagnose eye complications could have led to different results. Furthermore, differences in health care system [16] as well as in the study outcome definition across studies need to be considered; while we included any type of DMP, other study considered progression to photocoagulation treatment [17]. Temporal trends in the standard retinopathy diagnostic techniques and in the management of diabetes and hypertension, in the last decade, should also be considered as potential contributors to explain differences between studies. In relation to potential secular trends, we evaluated independently the incidence rates of DR and DMP in two calendar periods before and after the establishment of a widespread diabetic eye screening programme in the UK. The implementation of a national screening program started in 2003 [18] and the number of diabetic patients invited to screening has steadily been growing, reaching approximately 85% of diabetic patients in 2007 [19]. In our study we have observed that the IR of DR and DMP during first 2 years after diabetes diagnosis was two-fold higher for DR and three-fold higher for DMP diabetics diagnosed at the end of the study period (2006–2007) compared to those diagnosed in the initial years (2000–2001). Further, the possible differences of standard retinopathy diagnostic techniques used in UK at the beginning of the study period, such as direct ophthalmoscopy (known to be of low sensitivity [20]) versus two field digital retinal photography (the accepted method of screening since 2006 [18]) could have already been translated into improvements of early detection of eye complications in diabetic patients as shown already in the Liverpool Diabetic Eye Study [2], [3]. Also, although patients diagnosed with diabetes in 2000–2001 were comparable to those diagnosed in 2006–2007 in terms of diabetes treatment at diabetes onset (considered a proxy for severity of diabetes), type of diabetes, sex, and age, imbalances of other potential risk factors between these two groups might also contribute to the observed difference. Further research is required to clarify the issue.

In our study, a large proportion of DR and DMP cases were diagnosed shortly after diabetes diagnosis. This pattern suggests that, at least in some cases, the true onset of diabetes may have occurred before its initial clinical diagnosis, as described previously [21], [22], [23], which would be consistent with the fact that duration of diabetes is one of the most important determinants of ocular complications [2], [3], [24], [25].

The current study has several strengths. Importantly, our definition of DR and DMP was validated through review of PCPs' free text comments and response to a questionnaire. High confirmation rates were obtained, ensuring the internal validity of our results [10]. Results from our study can be extrapolated to the general population as THIN database has previously been shown to be representative of the UK general population [6]. The large cohort of diabetic patients captured has limited the possibility of chance findings occurring. Finally, our study provides an accurate view of the natural history of ocular complications of diabetes in newly identified diabetic patients in the last years. Our study has also some potential limitations. Imprecise ascertainment of the date of actual diabetes onset may have occurred in some patients. As previously mentioned, type 2 diabetes is often diagnosed late in the natural history of the disease [21], [22], [23]. This may have led to an underestimation of the true interval between the onset of diabetes and the occurrence of retinal complications. Also, the lack of detailed information of the DR characteristics in the patients' records precluded the analysis of progression from DR to DMP based on proliferative status or severity of DR. Finally, despite the large size of the study population, limited number of individuals with type 1 diabetes resulted in imprecise estimations of the cumulative incidence of DMP and DMO in this population.

In summary, in an incident population of diabetic patients in a UK primary care setting, we observed that close to 28% of patients developed retinopathy and close to 4% developed maculopathy (half were macular oedema) within 9 years of diabetes diagnosis. The incidence rate of DR and DMP during the first 2 years with diabetes was greater among patients diagnosed with diabetes in the last years of the period of study (2006–2007) than among those diagnosed earlier (2000–2001).

These data together with the reported increase of diabetes prevalence in the UK [11], [26] suggest that visual impairment due to diabetes complications continues to be a major public health issue. Additional analyses to evaluate risk factors associated with the occurrence of diabetic retinopathy under contemporary diabetes management protocols are needed to develop effective preventive strategies and treatment.
